# Prediction of SARS-CoV-2 hosts among Brazilian mammals and new coronavirus transmission chain using evolutionary bioinformatics

**DOI:** 10.1186/s44149-021-00020-w

**Published:** 2021-09-26

**Authors:** Luciano Rodrigo Lopes, Giancarlo de Mattos Cardillo, Natália Carvalho de Lucca Pina, Antonio Carlos da Silva Junior, Silvana Kertzer Kasinski, Paulo Bandiera-Paiva

**Affiliations:** 1 Bioinformatics and Bio-Data Science Division - Health Informatics Department, Universidade Federal de São Paulo – UNIFESP, Rua Botucatu 862 – Prédio Leal Prado (térreo), CEP: 04023-062, Sao Paulo [SP], SP Brasil; 2 Department of Neurology and Neurosurgery, Universidade Federal de São Paulo – UNIFESP, Sao Paulo [SP], SP Brasil

**Keywords:** SARS-CoV-2, Angiotensin-converting enzyme 2, Coronavirus, Brazilian mammals, White-tailed deer

## Abstract

Severe acute respiratory syndrome coronavirus (SARS-CoV) and SARS-CoV-2 are thought to transmit to humans via wild mammals, especially bats. However, evidence for direct bat-to-human transmission is lacking. Involvement of intermediate hosts is considered a reason for SARS-CoV-2 transmission to humans and emergence of outbreak. Large biodiversity is found in tropical territories, such as Brazil. On the similar line, this study aimed to predict potential coronavirus hosts among Brazilian wild mammals based on angiotensin-converting enzyme 2 (ACE2) sequences using evolutionary bioinformatics. Cougar, maned wolf, and bush dogs were predicted as potential hosts for coronavirus. These indigenous carnivores are philogenetically closer to the known SARS-CoV/SARS-CoV-2 hosts and presented low ACE2 divergence. A new coronavirus transmission chain was developed in which white-tailed deer, a susceptible SARS-CoV-2 host, have the central position. Cougar play an important role because of its low divergent ACE2 level in deer and humans. The discovery of these potential coronavirus hosts will be useful for epidemiological surveillance and discovery of interventions that can contribute to break the transmission chain.

**Supplementary Information:**

The online version contains supplementary material available at 10.1186/s44149-021-00020-w.

## Background

Severe acute respiratory syndrome coronavirus (SARS-CoV) and SARS-CoV-2 enter the host cell using surface (S) protein as a key component and via angiotensin-converting enzyme 2 (ACE2) receptor binding (Guruprasad 2020; Li et al. 2006). Changes in the structure of S protein can reduce its affinity for ACE2 receptor and thus can impair coronavirus infectivity (Li et al. 2005). Therefore, ACE2 could be a species-specific barrier that interferes with coronavirus cross-species transmission. However, mutations in genes coding S protein might be responsible for crossing the species-specific barrier and effective binding of SARS-CoV-2 to new hosts (Alexander et al. 2020; Li et al. 2005). Accordingly, S protein is the fastest-evolving protein responsible for SARS-CoV transmission from animals to humans (Song et al. 2005).

The transmission routes of SARS-CoV, Middle East respiratory syndrome coronavirus (MERS-CoV), and SARS-CoV-2 encompass a series of wild animals (Tiwari et al. 2020). Among these animals, bats are a broad reservoir of coronavirus (Wong et al. 2020). For instance, *Rhinolophus affinis*, an Asian bat, is a host for a coronavirus strain having high genetic similarity with SARS-CoV-2. Additionally, betacoronavirus strains, partially similar to SARS-CoV, were detected in neotropical bat species such as *A. lituratus*, *C. perspicillata* and *D. rotundus* (Brandão et al. 2008; Corman et al. 2013; Góes et al. 2016). The betacoronavirus strain was found in Brazilian territory that has 178 bat species (Nogueira et al. 2014), and might favor the spillover of coronavirus to new susceptible host species.

Bats are a potential reservoir for several human pathogenic viruses, including coronavirus (Calisher et al. 2006). However, they exhibit remarkable resilience without manifestation of diseases because of their unique immune system (Brook and Dobson 2015). The high population density of bats favors viral dispersion (Calisher et al. 2006). Several species of bats fly over long distances during seasonal migrations (Popa-Lisseanu and Voigt 2009), and might be responsible for further spread of viruses to geographically distant locations. Furthermore, the use of echolocation for navigation produced by a specialized larynx can result in high dispersion of droplets and aerosols, which also facilitates viral transmission (Calisher et al. 2006).

Although SARS-like-CoV and SARS-like-CoV-2 are widely spread in bats, direct bat-to-human transmission has no evidence (Lu et al. 2015; Wong et al. 2020). Therefore, an intermediate host might be involved in the transmission of coronavirus to humans by improving the affinity of S protein for ACE2 receptor (Wang et al. 2006). In addition, similarity of ACE2 between hosts seems to be an important fator for zoonotic transmission. In a previous study, our group reported that human ACE2 receptor showed low divergence compared with ACE2 receptor from civet or pangolin, both which are known as intermediate hosts for coronavirus outbreaks. However, human ACE2 receptor showed higher divergence than that of bat ACE2 receptor (Lopes et al. 2020). Intermediate divergences reported between human and civet/pangolin ACE2 might contribute to the transmission of SARS-CoV and SARS-CoV-2, respectively.

The increased contact between animals and humans has raised concerns about the emergence of new coronavirus outbreak. Zoonotic transmission of SARS-CoV-2 has been proved, however, the spillover of SARS-CoV-2 from humans to wild animals must be considered. Evidence within mink farms has indicated the transmission of SARS-CoV-2 between humans and mink and back to humans (Munnink et al. 2021). Moreover, feral cats that roam on and beyond the mink farms were determined to be infected by SARS-CoV-2. These cats were likely infected by SARS-CoV-2-infected minks (van Aart et al. 2021). Furthermore, owing to the detection of SARS-CoV-2 in zoo animals, human-to-wild animal transmission has also been discussed (McAloose et al. 2020; Singla et al. 2020). The close contact between human population infected with SARS-CoV-2 and wild mammals might be a potential threat for new coronavirus outbreak. Based on this assumption, we aimed to study ACE2 receptor in Brazilian wild mammals to predict potential hosts for coronavirus using evolutionary bioinformatics analyses.

## Results

We performed phylogenetic analysis including 34 ACE2 protein complete sequences, obtained from the NCBI Protein database (www.ncbi.nlm.nih.gov/protein/) using MrBayes V.3.2 (Ronquist et al. 2012). ACE2 sequences from 27 species of wild mammals found in Brazil, five known coronavirus hosts, *Homo sapiens* and *A. mississippiensis* (outgroup) were included in this study. ACE2 sequences from known SARS-CoV, MERS-CoV and SARS-CoV-2 hosts were included to determine the phylogenetic relationship between these animals and Brazilian wild mammals to predict potential hosts among them. The accession numbers of all sequences are listed in Supplementary Table 1. ACE2 protein sequences were aligned using MUSCLE (Edgar 2004). To construct a phylogenetic tree, the Jones-Taylor-Thornton model (Jones et al. 1992) with a gamma distribution for among-site rate variation (JTT + G model) was used, selected by the model test conducted in MEGA X (Kumar et al. 2018). Additional analysis of a phylogenetic tree of cervid species, including 11 mitochondrial cytochrome b amino acid sequences (listed in Supplementary Table 1), was performed using mtREV model (general reversible Markov model-REV for mitochondrial DNA-encoded proteins) (Adachi and Hasegawa 1996), selected by the model test conducted in MEGA X (Kumar et al. 2018). Bayesian phylogenetic trees were searched for one million generations with sampling every 100 generation until the standard deviation from split frequencies was less than 0.01. The parameters and the trees were summarized by wasting 25% of the samples obtained (burn-in). A consensus tree was constructed and was further used to determine the posterior probabilities. Phylogenetic trees were formatted using the FigTree v1.3.1 software (http://tree.bio.ed.ac.uk/software/figtree/). We further performed evolutionary divergent pairwise analysis based on the number of amino acid substitutions per site, using the MEGA X software (Kumar et al. 2018), and constructed a heatmap matrix to complement the phylogeny. To infer the evolutionary divergence, we used the ACE2 complete sequence-based alignment. We also used a concatenated alignment with four ACE2 regions containing residues involved in binding to S protein of SARS-CoV-2 (Supplementary file 1). ACE2 binding regions that encompass the key residues enabling interactions with SARS-CoV-2 S protein were based on previous studies (Shang et al. 2020; Brown et al. 2021).

**Fig. 1:**
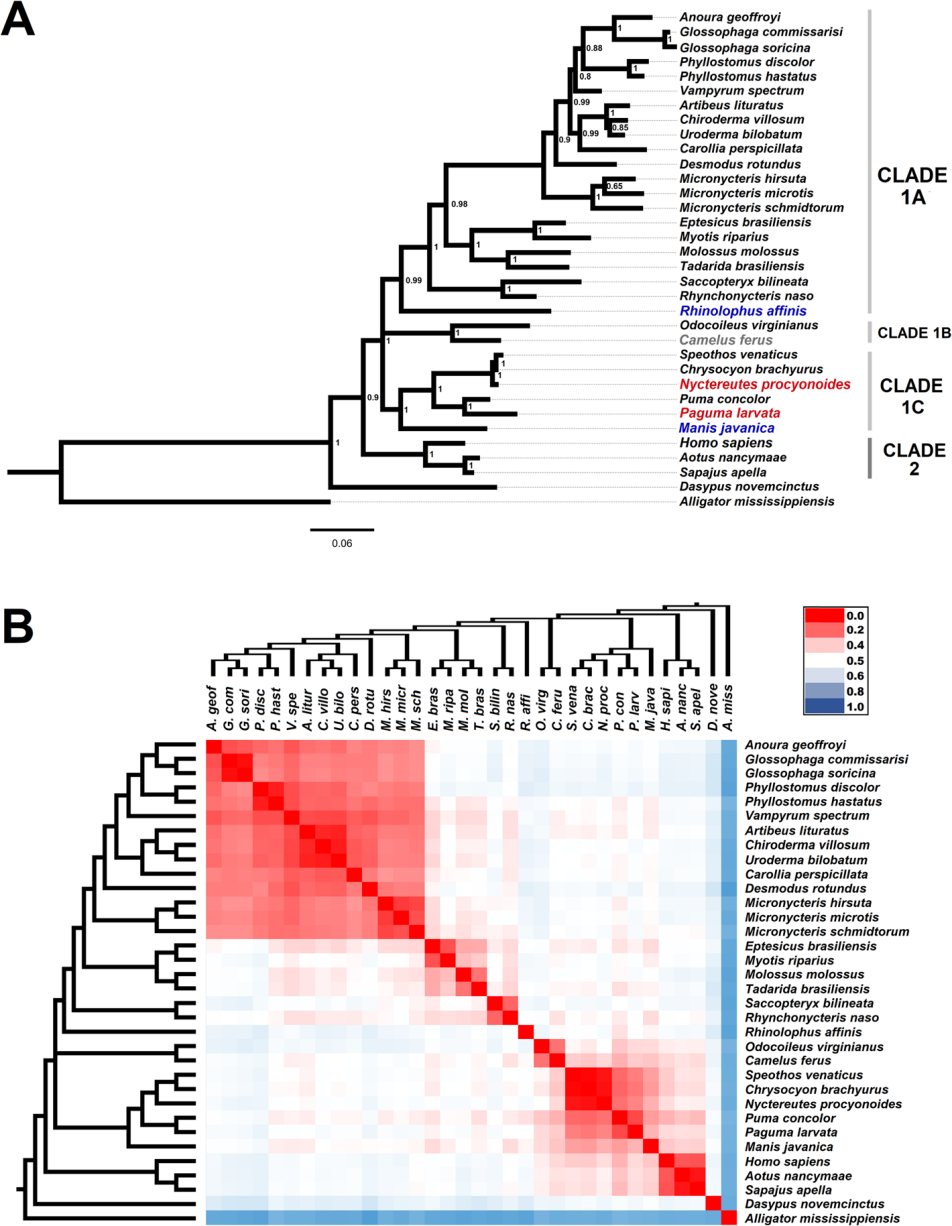
Evolutionary analysis based on angiotensin-converting enzyme 2 (ACE2). **a** Phylogenetic tree included 34 amino acid sequences. The species in blue represent the known severe acute respiratory syndrome coronavirus (SARS-CoV)-2 hosts, the species in gray represent the known MERS-CoV host, and the species in red represent the known SARS-CoV hosts. The numbers in the phylogenetic tree represent the posterior probability that are the confidence values from each clade (the higher confidence is 1). Scale bar indicates the number of substitutions/site for the trees. **b** Bayesian phylogenetic tree was based on ACE2 protein. ACE2 protein sequences were aligned and used to infer the evolutionary divergence values, represented in the matrix heatmap. The heatmap color gradient represents the evolutionary divergence based on the number of amino acid substitutions/site from a pairwise comparison between sequences, from low (red) to high (blue). Evolutionary divergence analyses were conducted using the JTT matrix-based model. The heatmap were constructed using the Microsoft Excel™ software

ACE2 phylogenetic tree (Fig. 1a), rooted in ACE2 of *A. mississippiensis*, showed two distinct clades. Clade 1 was clustered into three subclades: bats (A), ungulates (B), and felines/canids (carnivores) together with known SARS-CoV and SARS-CoV-2 hosts (C). Clade 2 is a small and outside clade composed of primates, encompassing humans. *D. novemcinctus* (armadillo) was placed distantly in the tree.

The phylogenetic tree of ACE2 sequence (Fig. 1a) revealed that clade 1C of SARS-CoV intermediate hosts nested with Brazilian wild carnivores. *Paguma larvata* (civet) is closely related to *Puma concolor* (cougar), whereas *Nyctereutes procyonoides* (raccoon dog) is closely related to *C. brachyurus* (maned wolf) and *Speothos venaticus* (bush dog). These close evolutionary relationships were confirmed by low divergence between SARS-CoV hosts and the indigenous carnivores (Fig. 1b and Table 1). *Manis javanica* (Malayan pangolin), the intermediate host of SARS-CoV-2, was placed together with cougar, maned wolf and bush dog in clade 1C and showed low divergence of ACE2 protein compared with these animals (Fig. 1b and Table 1), although it is not close to the Brazilian carnivores such as the civet and raccoon dog are.

**Table 1:** Angiotensin-converting enzyme 2 (ACE2) based-evolutionary divergence

Species	*A. geof*	*A. litu*	*C. pers*	*D. rotu*	*R. affi*	*O. virg*	*C. feru*	*S. vena*	*C. brac*	*N. proc*	*P. conc*	*P. larv*	*M. java*	*H. sapi*
*Anoura geoffroyi*														
*Artibeus lituratus*	0,112													
*Carollia perspicillata*	0,133	0,100												
*Desmodus rotundus*	0,125	0,107	0,125											
*Rhinolophus affinis*	0,283	0,266	0,262	0,289										
*Odocoileus virginianus*	0,304	0,272	0,273	0,302	0,273									
*Camelus ferus*	0,261	0,235	0,250	0,265	0,240	0,115								
*Speothos venaticus*	0,264	0,238	0,252	0,272	0,230	0,240	0,202							
*Chrysocyon brachyurus*	0,258	0,233	0,246	0,267	0,225	0,228	0,196	0,006						
*Nyctereutes procyonoides*	0,266	0,240	0,253	0,273	0,230	0,233	0,201	0,013	0,008					
*Puma concolor*	0,252	0,231	0,240	0,267	0,196	0,196	0,176	0,106	0,102	0,106				
*Paguma larvata*	0,269	0,251	0,257	0,287	0,220	0,214	0,201	0,128	0,120	0,123	0,074			
*Manis javanica*	0,254	0,231	0,242	0,244	0,219	0,215	0,193	0,159	0,154	0,163	0,155	0,178		
*Homo sapiens*	0,279	0,255	0,273	0,288	0,253	0,231	0,213	0,201	0,199	0,203	0,177	0,213	0,195	

ACE2-based analyses showed that humans are phylogenetically distant and evolutionarily divergent from bats. Bats were placed closer to the mammals of clades 1B and 1C than humans from clades 1B and 1C in the phylogenetic tree (Fig. 1a). However, the evolutionary divergence of humans from wild carnivores is lower than that from bats (Fig. 1b and Table 1). Among the indigenous carnivores, cougar showed lowest divergence from human ACE2 receptor (Table 1).

**Fig. 2:**
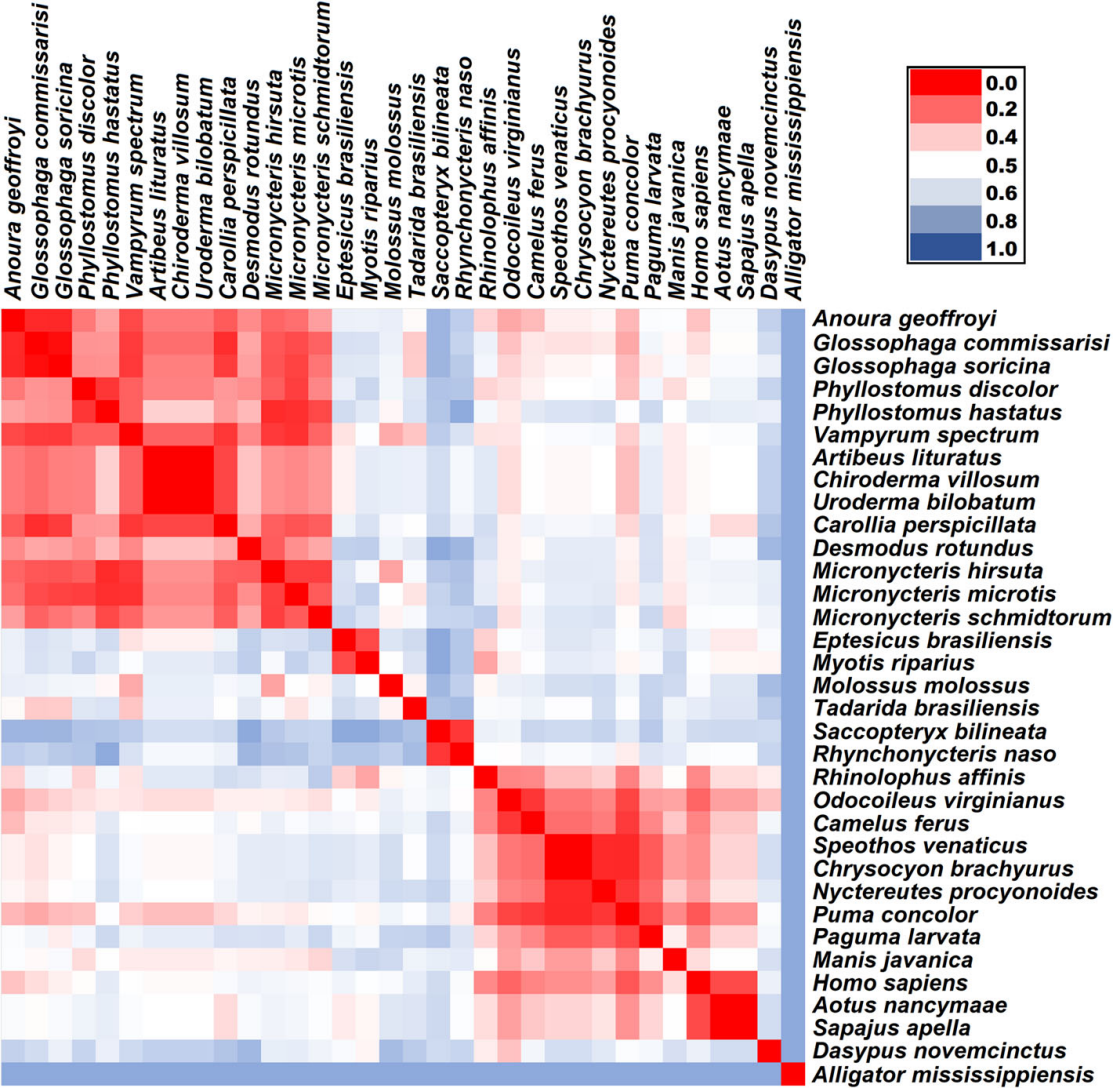
Heatmap matrix was based on four ACE2 binding regions that encompass the key residues enabling interactions with the SARS-CoV-2 S protein, detailed on Table 2 and Supplementary file 1. The ACE2 binding regions were concatened and used to infer the evolutionary divergence values, represented in the matrix heatmap. The evolutionary divergence values are available in the Supplementary Table 2. The heatmap color gradients represent the evolutionary divergence based on the number of amino acid substitutions/site from a pairwise comparison between sequences, from low (red) to high (blue). Evolutionary divergence analyses were conducted using the JTT matrix-based model. The heatmap were constructed using the Microsoft Excel™ software

An additional analysis targeting key residues based on the binding region of ACE2 protein, which are important regions in SARS-CoV-2 S protein-ACE2 interactions, revealed similarities between humans and other mammals (Table 2). Sequences from cougars had higher similiarity with those from humans; only three different ACE2 key residues involved in binding to S protein were observed between them. The similarity between cougar and human sequences was even higher than the similarities within the primate group. ACE2 key residues, involved in binding to S protein, from bush dog, maned wolf and white-tailed deer were also highly similar to human ACE2 key residues. Human ACE2 key residues in binding regions were more similar to those of indigenous mammals than to those of known coronavirus hosts, such as camel, civet and pangolin. Evolutionary divergent analysis based on ACE2 regions containing the residues that interface with SARS-CoV-2 S protein of different species also showed low divergence between human and cougar and white-tailed deer (Table 2 and Fig. 2). Moreover, ACE2 binding region from cougar and deer presented a low divergence to those from indigenous bats (Fig. 2 and Supplementary Table 2). These results reinforce the potential of Brazilian mammals to serve as susceptible hosts for SARS-CoV-2. Among Brazilian bats, ACE2 key residues from *Anoura geoffrey* had higher similarity in comparison with those of humans. However, *A. geoffrey* is a known host for alphacoronavirus, but there has been no reports of this bat species harbouring betacoronavirus (Corman et al. 2013).

**Table 2:** Comparison of Angiotensin-converting enzyme 2 (ACE2) key residues involved in binding to S protein of SARS-CoV-2

		ACE2 binding region 1																						ACE2 binding region 2						ACE2 binding region 3						ACE2 binding region 4					Different # of key residues	Evolutionary divergence estimates
Species	*	24*	25	26	27*	28*	29	30	31*	32	33	34*	35*	36	37*	38*	39	40	41*	42*	43	44	45*	*	80	81	82*	83*	84*	325*	326	327	328	329*	330*	353*	354*	355*	356	357*		
*Homo sapiens*	S	Q	A	K	T	F	L	D	K	F	N	H	E	A	E	D	L	F	Y	Q	S	S	L	L	A	Q	M	Y	P	Q	G	F	W	E	N	K	G	D	F	R	0/23	0
*Aotus nancymaae*	S	Q	A	K	T	F	L	D	K	F	N	H	E	A	E	D	L	F	**H**	**E**	**N**	S	L	L	A	Q	**T**	Y	P	Q	G	F	W	E	N	K	**Q**	D	F	R	4/23	0.157
*Sapajus apella*	S	Q	A	K	T	F	L	D	K	F	N	H	E	A	E	D	L	F	**H**	**E**	**N**	S	L	L	A	Q	**T**	Y	P	Q	G	F	W	E	N	K	**Q**	D	F	R	4/23	0.157
*Manis javanica*	S	**E**	A	K	T	F	L	**E**	K	F	N	**S**	E	A	E	**E**	L	**S**	Y	Q	S	S	L	**I**	A	**K**	**N**	Y	**Q**	Q	**T**	F	W	E	N	K	**H**	D	F	R	7/23	0.468
*Paguma larvata*	S	**L**	A	K	T	F	L	**E**	**T**	F	N	**Y**	E	A	**Q**	**E**	L	**S**	Y	Q	S	S	**V**	L	A	Q	**T**	Y	P	Q	G	F	W	E	N	K	G	D	F	R	7/23	0.303
*Puma concolor*	S	**L**	A	K	T	F	L	**E**	K	F	N	H	E	A	E	**E**	L	**S**	Y	Q	S	S	L	L	A	**K**	**T**	Y	P	Q	G	F	W	E	N	K	G	D	F	R	3/23	0.185
*Nyctereutes procyonoides*	S	**L**	**V**	**N**	T	F	L	**E**	K	F	N	**Y**	E	A	E	**E**	L	**S**	Y	Q	S	S	L	L	A	**K**	**T**	Y	P	Q	G	F	W	E	N	**R**	G	D	F	R	5/23	0.333
*Chrysocyon brachyurus*	S	**L**	**V**	K	T	F	L	**E**	K	F	N	**Y**	E	A	E	**E**	L	**S**	Y	Q	S	S	L	L	A	**K**	**T**	Y	P	Q	**E**	F	W	E	N	K	G	D	F	R	4/23	0.302
*Speothos venaticus*	S	**L**	**V**	K	T	F	L	**E**	K	F	N	**Y**	E	A	E	**E**	L	**S**	Y	Q	S	S	L	L	A	**K**	**T**	Y	P	Q	**E**	F	W	E	N	K	G	D	F	R	4/23	0.302
*Odocoileus virginianus*	S	Q	A	K	T	F	L	**E**	K	F	N	H	E	A	E	D	L	**S**	Y	Q	S	S	L	**M**	A	**K**	**T**	Y	**S**	Q	G	F	W	**D**	N	K	G	D	F	R	4/23	0.213
*Camelus ferus*	S	**L**	A	K	T	F	L	**E**	**E**	F	N	H	E	A	E	D	L	**S**	Y	Q	S	S	L	**T**	A	**K**	**T**	Y	P	Q	G	F	W	**D**	N	K	G	D	F	R	5/23	0.279
*Dasypus novemcinctus*	S	Q	A	**S**	T	F	L	**E**	**T**	F	N	**Q**	**Q**	A	E	**E**	L	**S**	**H**	Q	S	**A**	L	**M**	A	Q	**N**	**F**	**S**	**E**	G	F	W	**N**	N	K	G	D	F	R	10/23	0.624
*Rhinolophus affinis*	S	**R**	A	K	**I**	F	L	D	**N**	F	N	H	E	A	E	D	L	**S**	Y	Q	S	S	L	L	A	**K**	**N**	Y	P	**E**	G	F	W	**N**	N	K	G	D	F	R	6/23	0.291
*Rhynchonycteris naso*	**P**	Q	A	K	T	F	L	D	K	F	N	**Y**	E	A	E	**Q**	L	**S**	**F**	**E**	S	S	L	**R**	A	**K**	**A**	**F**	P	**E**	G	F	W	**K**	N	K	**N**	D	F	R	11/23	0.567
*Saccopteryx bilineata*	**P**	**E**	A	K	T	F	L	D	**R**	F	N	**Y**	E	A	E	**Q**	L	**S**	**F**	**E**	S	S	L	**R**	A	**K**	**D**	**F**	**S**	**E**	G	F	W	**K**	N	K	**N**	D	F	R	13/23	0.756
*Tadarida brasiliensis*	S	**E**	A	K	**I**	F	L	**Q**	**R**	F	N	**T**	E	A	E	**E**	L	**H**	**H**	Q	**N**	S	L	**H**	A	**K**	**R**	Y	P	Q	**E**	F	W	**N**	N	K	G	D	F	R	9/23	0.651
*Molossus molossus*	S	**K**	A	K	**I**	F	L	D	**N**	F	N	**I**	**R**	A	E	**E**	L	**H**	**H**	Q	S	S	L	**Q**	A	**K**	M	Y	P	Q	G	F	W	**D**	N	**N**	**N**	D	F	R	11/23	0.569
*Myotis riparius*	S	**K**	A	K	**I**	F	L	**E**	**N**	F	N	**S**	**K**	A	E	D	L	**S**	**H**	**E**	S	**A**	L	L	A	Q	**T**	Y	P	**A**	G	F	W	**N**	N	?	G	D	F	R	10/22	0.550
*Eptesicus brasiliensis*	S	**N**	A	**T**	**I**	F	L	**E**	**N**	F	N	**S**	E	A	E	D	L	**S**	**H**	**E**	S	**A**	L	L	A	Q	**T**	Y	P	**P**	G	F	W	**N**	N	K	**D**	D	F	R	10/23	0.602
*Micronycteris schmidtorum*	**T**	**E**	A	**R**	T	F	L	**E**	K	F	N	**A**	**K**	A	E	**E**	L	**Y**	**H**	**R**	S	S	L	?	?	?	?	?	?	Q	G	F	W	**D**	N	K	**K**	D	F	R	9/19	0.562
*Micronycteris microtis*	**T**	**E**	A	**R**	**K**	F	L	**E**	**A**	F	N	**T**	E	A	E	**E**	L	**Y**	**H**	Q	S	S	L	L	A	**K**	?	?	?	Q	G	F	W	**D**	N	K	**K**	D	F	R	9/20	0.582
*Micronycteris hirsuta*	**T**	**E**	A	**R**	T	F	L	**E**	**N**	F	N	**T**	**K**	A	E	**E**	L	**Y**	**H**	Q	S	S	L	L	A	**K**	?	?	?	Q	G	F	W	**D**	N	**N**	**K**	D	F	R	10/20	0.595
*Desmodus rotundus*	**T**	**E**	A	**R**	T	F	L	**E**	**N**	F	N	**T**	E	A	E	**E**	**W**	F	Y	Q	**N**	S	L	**I**	A	**K**	**T**	Y	P	Q	G	F	W	**D**	N	**N**	**K**	D	F	R	10/23	0.609
*Carollia perspicillata*	**T**	**E**	A	**R**	T	F	L	**E**	K	F	N	**T**	E	A	E	**E**	L	**Y**	**H**	**E**	**L**	S	L	L	A	**K**	**A**	Y	P	Q	G	F	W	**D**	N	K	**N**	D	F	R	9/23	0.568
*Uroderma bilobatum*	**A**	**D**	A	**R**	T	F	L	**E**	K	F	N	**T**	E	A	E	**E**	L	**Y**	Y	**E**	S	**A**	L	L	A	**K**	**A**	Y	P	Q	G	F	W	**D**	N	K	**N**	D	F	R	8/23	0.528
*Chiroderma villosum*	**A**	**D**	A	**R**	T	F	L	**E**	K	F	N	**T**	E	A	E	**E**	L	**Y**	Y	**E**	S	**A**	L	L	A	**K**	**A**	Y	P	Q	G	F	W	**D**	N	K	**N**	D	F	R	8/23	0.528
*Artibeus lituratus*	**A**	**D**	A	**R**	T	F	L	**E**	K	F	N	**T**	E	A	E	**E**	L	**Y**	Y	**E**	S	**A**	L	L	A	**K**	**A**	Y	P	Q	G	F	W	**D**	N	K	**N**	D	F	R	8/23	0.528
*Vampyrum spectrum*	**T**	**E**	A	**R**	**I**	F	L	**E**	**N**	F	N	**T**	E	A	E	**E**	L	**Y**	**H**	Q	S	S	L	L	A	**K**	**A**	Y	P	Q	G	F	W	**D**	N	K	**N**	D	F	R	10/23	0.545
*Phyllostomus hastatus*	**T**	**E**	A	**R**	**K**	F	L	**E**	**N**	F	N	**N**	**K**	A	E	**E**	L	**Y**	**H**	Q	S	S	L	L	A	**K**	**N**	Y	**S**	Q	G	F	W	**D**	N	K	**K**	D	F	R	12/23	0.661
*Phyllostomus discolor*	**T**	**D**	A	**R**	**K**	F	L	**E**	**N**	F	N	**N**	E	A	E	**E**	L	**Y**	Y	Q	S	S	L	L	A	**K**	**N**	Y	P	Q	G	F	W	**D**	N	K	**K**	D	F	R	9/23	0.531
*Glossophaga soricina*	**T**	**E**	A	**R**	T	F	L	**E**	**T**	F	N	**T**	E	A	E	**E**	L	**Y**	**H**	Q	**R**	S	L	L	A	**K**	**A**	Y	P	Q	G	F	W	**D**	N	K	G	D	F	R	8/23	0.506
*Glossophaga commissarisi*	**T**	**E**	A	**R**	T	F	L	**E**	K	F	?	**T**	E	A	E	**E**	L	**Y**	**H**	Q	**R**	S	L	L	A	**K**	**A**	Y	P	Q	G	F	W	**D**	N	K	G	D	F	R	7/23	0.467
*Anoura geoffroyi*	**T**	**E**	A	**R**	T	F	L	**E**	**N**	F	N	**T**	E	A	E	D	L	**Y**	Y	Q	**R**	S	L	L	A	**K**	**A**	Y	P	Q	G	F	W	**D**	N	K	G	D	F	R	6/23	0.412

? character represents the missing data

* symbols indicate the ACE2 residues that interact with SARS-CoV-2 S protein

Our results, based on ACE2 analysis, revealed a close relationship and low divergence between the ungulates *Camel ferus* (camel), a known MERS-CoV host, and *Odocoileus virginianus* (white-tailed deer), a host susceptible to SARS-CoV-2 infection (Fig. 1 and Table 1). Additionally, the key residues in binding region of ACE2 protein of ungulates had few differences in comparison with those of human ACE2 (Table 2 and Fig. 2). MERS-CoV transmission from camels to humans requires close exposure to be effective (Han et al. 2016). In this context, humans also come in close contact with white-tailed deer because of their hunting and use as livestock (McShea 2012). SARS-CoV-2 transmission in farmed animals (American mink) has already been reported (Munnink et al. 2021). Furthermore, deer have high population density and are broadly distributed across all American continents, that are next to urban areas. For controling the population density of deer, their hunting is considered legal in some territories (McShea 2012). In northern Brazil, white-tailed deer are hunted and their meat is consumed by quilombola communities (de Figueiredo et al. 2016).

**Fig. 3:**
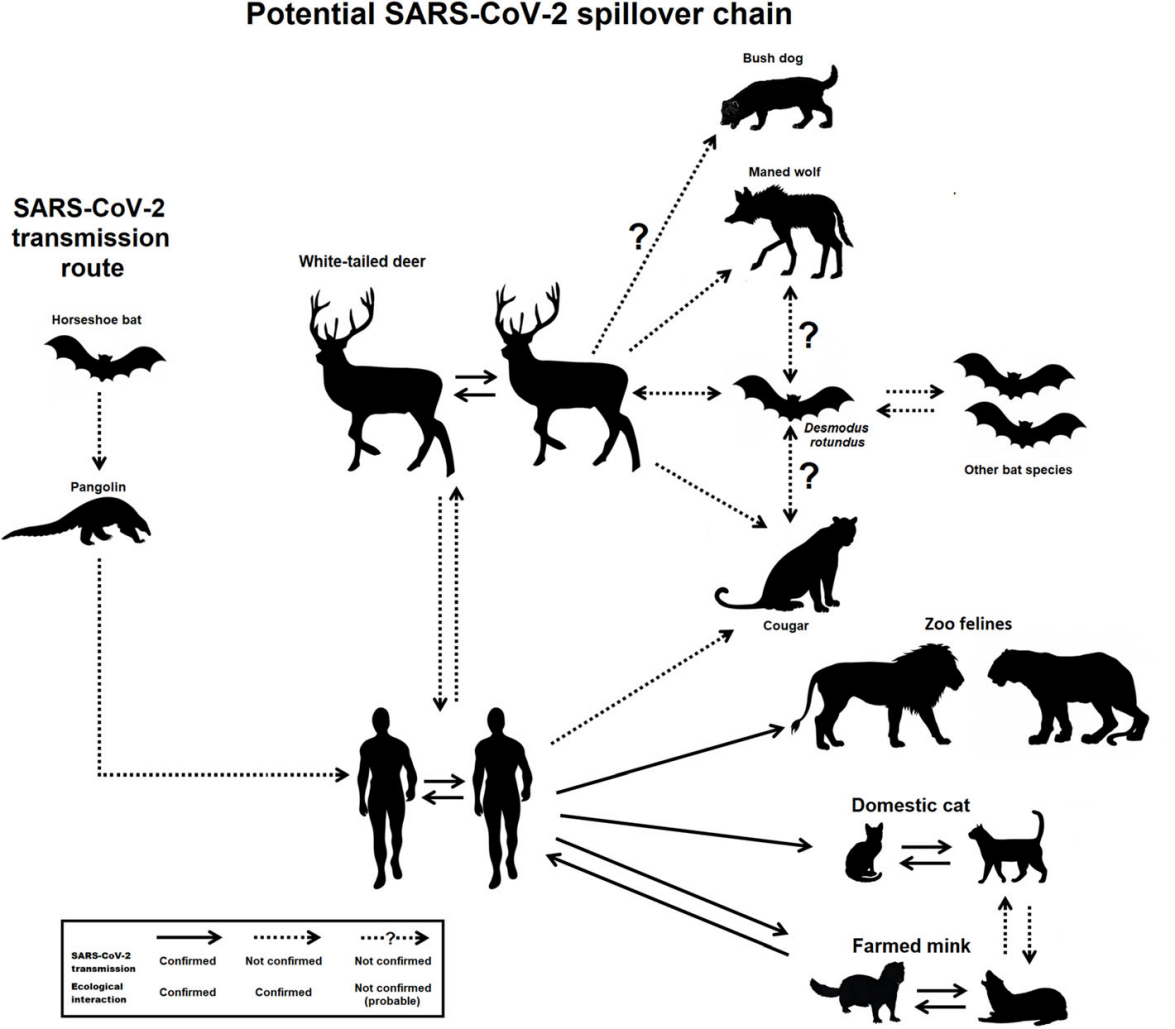
Diagram displaying the potential transmission chain of severe acute respiratory syndrome coronavirus (SARS-CoV)-2 among humans and wild animals. Known SARS-CoV-2 hosts (horseshoe bat and pangolin), susceptible hosts confirmed by previous studies (white-tailed deer, mink, domestic cat, and zoo felines) and potential SARS-CoV-2 hosts (cougar, maned wolf and bush dog) predicted by bioinformatics analysis were included. (From left to right) Horseshoe bats (*Rhinolophus* genus), the main animal reservoir of SARS-CoV-2 (SARS-like-CoV) and Malayan pangolin, the SARS-CoV-2 intermediate host. White-tailed deer are susceptible to SARS-CoV-2 infection and are preyed by cougars, a potential SARS-CoV-2 host predicted by this study. SARS-CoV-2 may transmit from humans to cougars, considering that lions and tigers were infected by SARS-CoV-2 in the zoo. Maned Wolf, a potential SARS-CoV-2 host, can prey on other deer species. Bush dog is likely to prey on deer, but evidence is lacking. *D. rotundus*, a vampire bat, is a SARS-like-CoV host that can feed on white-tailed deer and other deer species. Moreover, *D. rotundus* could spread coronavirus to other bat species or even to cougar, maned wolf or bush dog, although there is no scientific confirmation

Based on the close relationship between humans and white-tailed deer, and considering their susceptibility to SARS-CoV-2 infection, we proposed a scenario that represents the transmission chain of SARS-CoV-2, including the potential hosts predicted by our evolutionary bioinformatics analyses and considering the ecological features of the wild mammals (Fig. 3). White-tailed deer have intricate relationships with other wild animals and humans. For instance, white-tailed deer (and other deer species) have been frequently preyed on by cougars (Cooley et al. 2008; McShea 2012). Both the species share a large spatial area on the American continent. In some locations, cougars appear to primarily subsist on white-tailed deer during winter (Cooley et al. 2008). This situation could favor the transmission of SARS-CoV-2 from deer to cougar. In addition, our results showed low ACE2 evolutionary divergence between deer and cougar (Table 1), which may further contribute to potential transmission. SARS-CoV-2-infected wild felines from zoos (McAloose et al. 2020) and domestic cats (Halfmann et al. 2020) may contribute to the SARS-CoV-2 transmission hypothesis of the cougars being infected by SARS-CoV-2. SARS-CoV-2 spillover from humans to cougars is possible because of substantial conflicts between the two in farming frontiers (de Souza et al. 2018; Palmeira et al. 2015).

Additional ecological interactions involving white-tailed deer place these animals at the center of potential transmission chain (Fig. 3). A study reported that *D. rotundus* feed on white-tailed deer (Sánchez-Cordero et al. 2011). The high abundance of deer may constitute an important feed resource for vampire bats. The susceptibility of *D. rotundus* toward SARS-like-CoV infections indicates that vampire bat might be infected by SARS-CoV-2 and transmit the virus to other animals. *D. rotundus* was also observed feeding on another cervid species, red brocket deer (*Mazama americana*) (Zortéa et al. 2018). In addition, these South American cervid are preying upon by maned wolf (Juarez and Marinho-Filho 2002) and by bush dog, according to the observation of individuals living next to wild habitat (Beisiegel and Ades 2002). Although the susceptibility of red brocket deer to SARS-CoV-2 has not yet been confirmed, all cervid species should be considered potential hosts for SARS-CoV-2 because of the close evolutionary relationship between these animals and white-tailed deer (Supplementary Fig. 1).

Taken together, connections between SARS-CoV-2 susceptible hosts with potential hosts comprise a complex network and hence might contribute to SARS-CoV-2 transmission and emergence of new coronavirus strains. The history of SARS shows that coronavirus presents high variability, potential to infect wildlife and rapid evolution to adapt in new susceptible hosts, thus becoming a threat to humans (Gong and Bao 2018). SARS-like-CoV strains, frequently found in wildlife after the end of the SARS outbreak, can be implicated in SARS-CoV-2 origin (Zhang et al. 2020). High mutation rate and worldwide spread of SARS-CoV-2 contribute to the emergence of new variants (Benedetti et al. 2020). Thus, high mutation rates of SARS-CoV-2 may favor virus transmission in susceptible wild hosts.

Considering ACE2 receptor as a transmission barrier that interferes with the coronavirus spillover to humans, a low divergence between ACE2 receptors of susceptible hosts may favor cross-species transmission. The close evolutionary relationship between the known coronavirus intermediate hosts and Brazilian mammals based on ACE2 sequences showed that other hosts might be also be involved in the chain. Our results suggest that cougar, maned wolf, bush dog, and white-tailed deer, as well as their relative species, are potential hosts for SARS-CoV-2 transmission. We focused on white-tailed deer as a susceptible SARS-CoV-2 host because of their increased population, wide distribution, and close contact with humans and other potential hosts such as cougars. In this context, white-tailed deer and cougar (prey and predator) could be involved in coronavirus transmission together with tropical bats susceptible to betacoronavirus. The central role of white-tailed deer during SARS-CoV-2 outbreak, as well camels during MERS outbreak, can make them important coronavirus reservoirs. However, these results must be confirmed through animal experiments and studies to detect coronavirus strains and assess the ability of these animals to serve as susceptible hosts.

In Brazil, SARS-CoV-2 infections have been rapidly spreading, since the beginning of the coronavirus disease 2019 (COVID-19) pandemic, favoring the emergence of new variants (da Silva and Pena 2021). Thus, a high incidence of SARS-CoV-2 cases in populations that live in proximity to wild habitats, which harbor a large diversity of mammals, can be an additional and critical scenario of new emerging coronavirus strains in Brazilian territories. Genomic surveillance should be widely implemented to identify new variants and establish measures to control SARS-CoV-2 transmission. However, in the context of Brazil, SARS-CoV-2 surveillance has been slow (da Silva and Pena 2021). The discovery of potential hosts for coronaviruses is essential for epidemiological surveillance. Moreover, coronavirus transmission chain can be broken using effective intervention if all players involved in the chain are identified. Therefore, the surveillance of SARS-CoV-2 in domestic or wild animals, specially those with low divergence in the ACE2 receptor when compared to that of humans, should be broadly applied. Brazil should join forces to promote multi-sectoral responses, reducing the impact of the SARS-CoV-2 pandemic and consequently preventing the emergence of new coronavirus outbreaks.

## Supplementary Information

**Additional file 1: Supplementary Table 1.** Protein accession numbers.

**Additional file 2. **ACE2 key residues interfacing with SARS-CoV-2 S protein.

**Additional file 3: Supplementary Table 2.** Estimates of evolutionary divergence between ACE2 regions that encompass the key residues enabling interactions with the SARS-CoV-2 S protein. The number of amino acid substitutions per site between sequences are shown. Evolutionary analyses were performed using MEGA X software. Analyses were conducted using the JTT matrix-based model. The rate variation among sites was modeled with a gamma distribution. This analysis involved 34 amino acid sequences aligned using MUSCLE. All ambiguous positions were removed for each sequence pair (pairwise deletion option). A total of 40 positions were included in the final dataset.

**Additional file 4: Supplementary Figure 1.** Bayesian tree based on 11 cervid mitochondrial cytochrome b amino acid sequences. Phylogeny was performed using MrBayes 3.2.v. and the mtREV+G. Values of posterior probabilities are shown at the nodes of interest. The scale bar indicates the number of substitutions/site for the trees. Phylogenetic tree was formatted using the FigTree v1.3.1 software (http://tree.bio.ed.ac.uk/software/figtree/).

## Data Availability

All the data obtained from the NCBI Protein and NCBI GenBank databases included in this research study presented accession code/numbers for research or reanalysis.

## Abbreviations

ACE2: Angiotensin-converting enzyme 2
COVID-19: Coronavirus disease 2019
JTT: Jones-Taylor-Thornton
MERS: Middle East respiratory syndrome coronavirus
MERS-CoV: Middle East respiratory syndrome coronavirus coronavirus
mtREV: General reversible Markov model for mitochondrial DNA-encoded proteins
NCBI: National Center for Biotechnology Information
S: surface
SARS: Severe acute respiratory syndrome
SARS-CoV: Severe acute respiratory syndrome coronavirus
SARS-CoV-2: Severe acute respiratory syndrome coronavirus 2
