# Supplementary figures and images for: Prediction of SARS-CoV-2 hosts among Brazilian mammals and new coronavirus transmission chain using evolutionary bioinformatics
Source: Anim Dis. 2021 Sep 26;1(1):20. doi: 10.1186/s44149-021-00020-w (PMC8475823; doi:10.1186/s44149-021-00020-w)

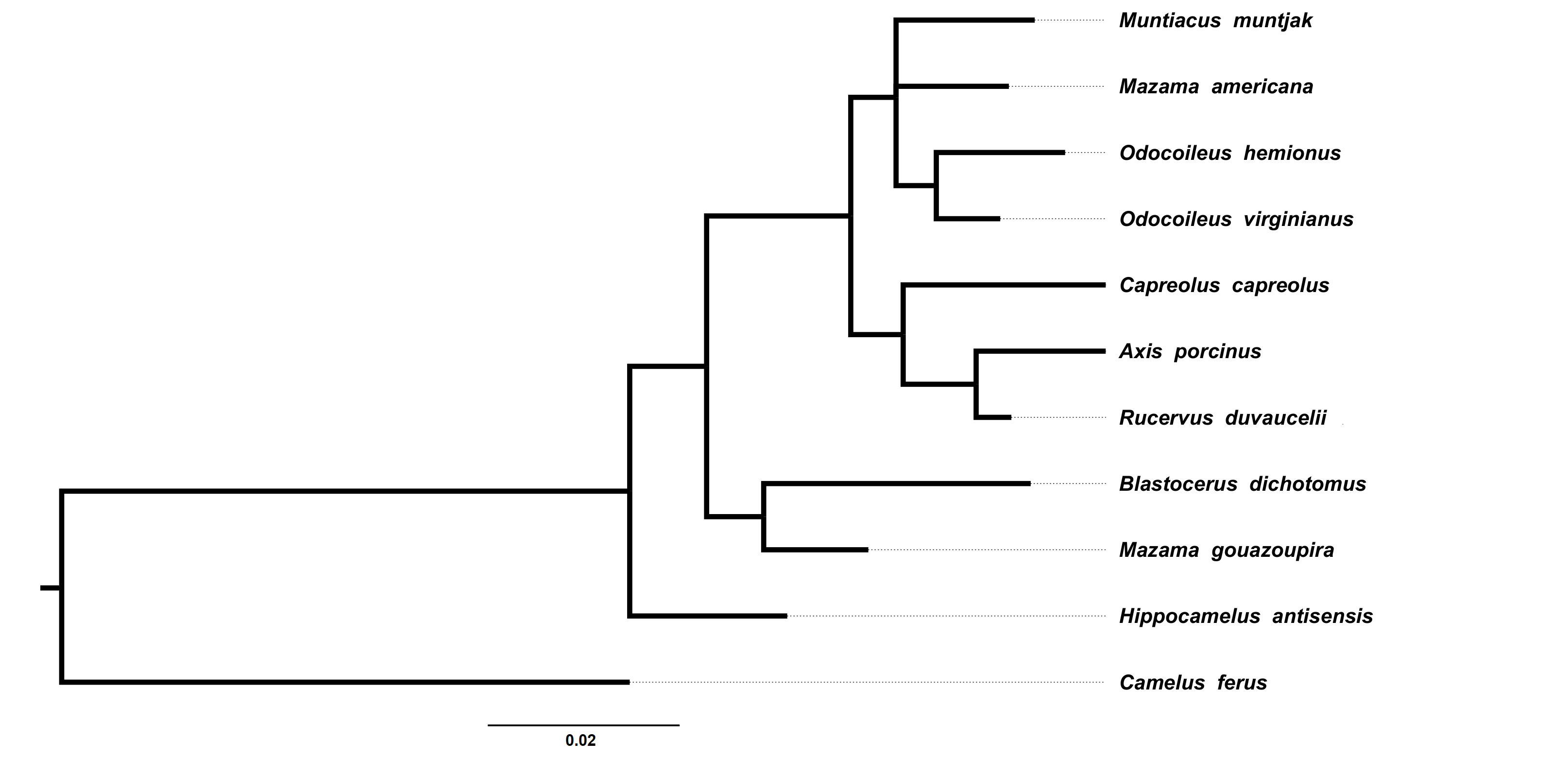

Supplement: Supplementary file 4 — Additional file 4: Supplementary Figure 1. Bayesian tree based on 11 cervid mitochondrial cytochrome b amino acid sequences. Phylogeny was performed using MrBayes 3.2.v. and the mtREV+G. Values of posterior probabilities are shown at the nodes of interest. The scale bar indicates the number of substitutions/site for the trees. Phylogenetic tree was formatted using the FigTree v1.3.1 software (http://tree.bio.ed.ac.uk/software/figtree/). [file 44149_2021_20_MOESM4_ESM.tif]
